# Current progress in perioperative chemotherapy for biliary tract cancer

**DOI:** 10.1002/ags3.12691

**Published:** 2023-05-16

**Authors:** Tatsuya Ioka, Yoshitaro Shindo, Makoto Ueno, Hiroaki Nagano

**Affiliations:** ^1^ Department of Oncology Center Yamaguchi University Hospital Ube Japan; ^2^ Department of Gastroenterological, Breast, and Endocrine Surgery Yamaguchi University Graduate School of Medicine Ube Japan; ^3^ Department of Gastroenterology, Hepatobiliary and Pancreatic Medical Oncology Division Kanagawa Cancer Center Yokohama Japan

**Keywords:** ampullary cancer, biliary tract cancer, cholangiocarcinoma, gallbladder cancer, perioperative chemotherapy

## Abstract

Biliary tract cancer (BTCs) is a heterogeneous malignancy divided into cholangiocarcinoma, gallbladder cancer, and ampullary cancer. Due to little or no symptoms, most patients with BTCs are diagnosed with unresectable or metastatic disease. Only 20%–30% of all BTCs are suitable for potentially resectable diseases. Although radical resection with a negative surgical margin is the only potentially curative method for BTCs, most patients develop postoperative recurrence, which is associated with poor prognosis. Therefore, perioperative treatment is necessary to improve survival. There are very few randomized phase III clinical trials of perioperative chemotherapy due to the relative rarity of BTCs. Adjuvant chemotherapy with S‐1 for patients with resected BTC significantly increased overall survival compared with upfront surgery in a recent ASCOT trial. In East Asia, S‐1 is currently considered the standard adjuvant chemotherapy, while capecitabine may still be used in other areas. Since then, our phase III trial (KHBO1401), gemcitabine and cisplatin plus S‐1 (GCS) has become the standard chemotherapy for advanced BTCs. GCS not only improved overall survival but demonstrated a high response rate. The efficacy of GCS as a preoperative neoadjuvant chemotherapy for resectable BTCs has been investigated in a randomized phase III trial (JCOG1920) in Japan. In this review, we summarize the current and ongoing clinical trials focusing on adjuvant and neoadjuvant chemotherapy for BTCs.

## Abstract 2

Although surgical resection is the only curative treatment option for biliary tract cancers (BTCs), the recurrence rate is high. Hence, effective perioperative chemotherapy needs to be developed to improve poor prognosis. The purpose of this review was to summarize the current and ongoing clinical trials focusing on adjuvant and neoadjuvant chemotherapy for BTCs.

## 1 INTRODUCTION

Biliary tract cancer (BTC) is a heterogeneous malignancy that includes cholangiocarcinoma, gallbladder cancer, and ampullary cancer.^1^, ^2^ According to the anatomical site, cholangiocarcinoma is subclassified into intrahepatic, perihilar, and distal cholangiocarcinoma.^3^, ^4^, ^5^ BTCs are relatively rare tumors. In Western countries, BTCs account for ~3% of all gastrointestinal tumors. In contrast, they are common in Japan, Southeast Asia, and India.^5^, ^6^, ^7^, ^8^, ^9^ In Japan, BTC is the sixth leading cause of cancer‐related deaths, accounting for ~18 000 deaths annually.^10^

Only 20%–30% of BTCs are resectable at diagnosis. Most patients with BTCs are diagnosed with unresectable or metastatic disease.^11^, ^12^, ^13^, ^14^, ^15^ The combination of gemcitabine (GEM) and cisplatin therapy (GC) as the first‐line standard treatment for advanced BTC was compared with GEM monotherapy in the ABC‐02 trial.^16^ In addition to GC, GEM, and S‐1, oral fluoropyrimidine prodrug, therapy (GS), and GC and S‐1 therapy (GCS) are also considered first‐line standard chemotherapy options, and are commonly used in Japan following previous phase III studies.^17^, ^18^, ^19^ Despite advances in chemotherapy, there are no standard second‐line treatment options for BTCs.^20^, ^21^, ^22^ Therefore, the median overall survival (OS) remains dismal at ~1 y.^16^, ^17^, ^18^

Surgical resection is the only curative treatment available for BTCs. Recent advances in surgical techniques, such as concomitant vascular resection and perioperative management, have increased resectability and improved patient outcomes.^23^, ^24^, ^25^, ^26^, ^27^ Positive surgical margins, lymph node metastasis, and venous invasion are prognostic factors for predicting OS and disease‐free survival (DFS).^28^, ^29^, ^30^ Despite curative resections, most patients develop postoperative recurrences, and the prognosis remains unsatisfactory, with a 5‐y survival rate of <50%.^31^, ^32^, ^33^, ^34^, ^35^ To improve long‐term outcomes after surgery, perioperative chemotherapy has been investigated, although surgical resection alone remains the first‐line therapy for patients with resectable BTC. In this review, we summarize the current and ongoing clinical trials that focused on perioperative chemotherapy for BTCs. A literature search was carried out using the PubMed database. The following terms were used in combination: “biliary tract cancer” and (“adjuvant therapy” or “neoadjuvant therapy”) and “clinical trial.” Original articles published in English were included. Relevant articles or clinical trials identified through manual searching of reference lists were also included.

## 2 ADJUVANT CHEMOTHERAPY

Surgical resection is the only curative treatment option for BTCs, but the recurrence rate is high. Hence, effective adjuvant therapy needs to be developed to improve a poor prognosis. Due to the relative rarity of BTCs, evidence for adjuvant chemotherapy is based on a few phase II clinical trials and retrospective analyses.^36^, ^37^, ^38^, ^39^ Between 1986 and 1992, the first randomized controlled trial was conducted to evaluate adjuvant chemotherapy with mitomycin C and fluorouracil (MF group) vs surgery alone (control group) in patients with pancreatic carcinoma (*n* = 158), bile duct carcinoma (*n* = 118), gallbladder carcinoma (*n* = 112), or ampullary cancer (*n* = 48).^40^ No statistically significant difference was observed in the 5‐y OS and 5‐y DFS rates between patients with pancreatic carcinoma, bile duct carcinoma, or ampullary cancer. Although the 5‐y OS rate of the MF group (26.0% vs 14.4%, *P =* 0.0367) and the 5‐y DFS rate (20.3% vs 11.6%, *P =* 0.0210) in gallbladder carcinoma improved, no significant improvement was demonstrated in the intent‐to‐treat analysis. Over the last decade, several adjuvant chemotherapy regimens have been investigated in randomized phase III trials (Table 1).

**TABLE 1:** Randomized phase III trials of adjuvant chemotherapy for resected biliary tract cancer.

Study (year)	Country or region	Tumor site	Treatment	Number of patients	Median OS (mo)	HR (95% CI)	*P*‐value
ESPAC‐3 (2012)	Europe	ECC, AmpC	5‐FU + FA or GEM vs observation	428	43.1 vs 35.2	0.86 (0.66–1.11)	0.25
BCAT (2018)	Japan	ECC	GEM vs observation	225	62.3 vs 63.8	1.01 (0.70–1.45)	0.964
PRODIGE 12‐ ACCORD 18 (2019)	France	ICC, ECC, GBC	GEMOX vs observation	196	75.8 vs 50.8	1.08 (0.70–1.66	0.74
BILCAP (2019)	United Kingdom	ICC, ECC, GBC	Capecitabine vs observation	447	51.1 vs 36.4	0.81 (0.63–1.04)	0.097
ASCOT (2023)	Japan	ICC, ECC, GBC, AmpC	S‐1 vs observation	440	3‐y OS 77.1% vs 67.6%	0.69 (0.51–0.94)	0.008

Abbreviations: 5‐FU, 5‐fluorouracil; AmpC, ampulla of Vater carcinoma; CI, confidence interval; ECC, extrahepatic cholangiocarcinoma; FA, folinic acid; GBC, gallbladder carcinoma; GEM, gemcitabine; GEMOX, gemcitabine plus oxaliplatin; HR, hazard ratio; ICC, intrahepatic cholangiocarcinoma; OS, overall survival.

### 2.1 The ESPAC‐3 trial

This phase III study compared surgery alone with 5‐FU plus folinic acid or gemcitabine monotherapy in 428 patients with periampullary region cancer (mainly ampullary carcinoma [*n* = 297] and extrahepatic cholangiocarcinoma [*n* = 96]).^41^ The primary outcome was OS in the treatment group compared with that in the surgery alone group. Median OS was 35.2 mo in the surgery alone group and 43.1 mo in the chemotherapy group (hazard ratio [HR] 0.86, 95% confidence interval [CI]; 0.66–1.11, *P =* 0.25), showing no superiority of chemotherapy. However, the sensitivity analysis adjusted for other prognostic factors, such as age, tumor grade, and lymph node status showed a significant prognostic benefit in the chemotherapy group (HR 0.75, 95% CI; 0.57–0.98, *P =* 0.03) and in the GEM group (HR 0.70, 95% CI; 0.51–0.97, *P =* 0.03), compared to the surgery alone group. Of note, no OS benefit was demonstrated in the intent‐to‐treat (ITT) analysis, and the data were underpowered and, thus, significance could not be concluded.

### 2.2 The BCAT trial

This phase III trial in Japan compared surgery alone with GEM monotherapy in 225 patients with resected perihilar and distal cholangiocarcinoma.^42^ The primary outcome was OS. The median OS was 63.8 mo in the surgery alone group, compared with 62.3 mo in the GEM group (HR 1.01, 95% CI; 0.70–1.45, *P =* 0.964). The median relapse‐free survival (RFS) was 36.0 mo in the GEM group and 39.9 mo in the surgery alone group (HR 0.93, 95% CI; 0.66–3.32, *P =* 0.693). No prognostic benefits were observed in the GEM group.

### 2.3 The PRODIGE 12/ACCORD‐18 trial

This phase III trial in France compared surgery alone with the combination of GEM and oxaliplatin (GEMOX) in 196 patients with resected BTCs, excluding ampullary carcinoma.^43^ This study included ~45% intrahepatic cholangiocarcinomas. The primary endpoint was RFS. Median RFS was 30.4 mo in the GEMOX group vs 30.4 mo in the surgery alone (HR 0.88, 95% CI; 0.62–1.25, *P =* 0.48). The median OS was 75.8 mo in the GEMOX group vs 50.8 mo in the surgery alone group (HR 1.08, 95% CI; 0.70–1.66, *P =* 0.74), showing no superiority of GEMOX. In the subgroup analyses, GEMOX demonstrated significantly shorter RFS (HR 2.56, 95% CI; 1.04–6.32, *P =* 0.034) and OS (HR 3.39, 95% CI; 1.17–9.83, *P =* 0.017) in patients with gallbladder cancer, although the number of patients in these subgroups was small. The effect of chemotherapy may have been influenced by biological differences, depending on the primary site.^44^ In the multivariate analysis, positive surgical margin (HR 1.99, 95% CI; 1.13–3.50, *P =* 0.017), nodal positive (HR 2.31, 95% CI; 1.53–3.50, *P <* 0.001), and microvascular invasion (HR 2.33, 95% CI; 1.55–3.51, *P <* 0.001) were independently associated with worse RFS.

### 2.4 The BILCAP trial

This phase III trial in the UK compared surgery alone with capecitabine in patients with resected cholangiocarcinoma and gallbladder cancer.^45^ The primary endpoint was OS. In an ITT analysis of 447 patients, the median OS was 51.1 mo in the capecitabine group and 36.4 mo in the surgery alone group (HR 0.81, 95% CI; 0.63–1.04, *P =* 0.097), indicating a longer but not superior outcome. However, a per‐protocol analysis of 430 patients, excluding patients who were ineligible at enrollment or who did not receive capecitabine, showed that capecitabine significantly prolonged prognosis compared to surgery alone (53 mo vs 36 mo, HR 0.75, 95% CI; 0.58–0.97, *P =* 0.028). In an ITT analysis, the median RFS was 24.4 mo in the capecitabine group and 17.5 mo in the surgery alone group (HR 0.75, 95% CI; 0.58–0.98, *P =* 0.033). In a per‐protocol analysis, the median RFS was 25.9 mo in the capecitabine group and 17.4 mo in the surgery alone group (HR 0.70, 95% CI; 0.54–0.92, *P =* 0.0093). Although the OS in the ITT analysis did not reach statistical significance, the OS in the per‐protocol analysis significantly improved. Due to the significant effect of capecitabine in improving prognosis, the current American Society of Clinical Oncology guidelines recommend capecitabine for 6 mo as adjuvant chemotherapy following surgery for patients with resected biliary tract cancer.^11^

### 2.5 The ASCOT trial

Recently, a randomized phase III study of S‐1 vs observation in patients with curatively resected BTCs (JCOG1202: ASCOT) was published.^46^ Between September 2013 and June 2018, 440 patients were randomly assigned to receive either adjuvant S‐1 (*n* = 218) or observation (*n* = 222). The primary endpoint was the superiority of adjuvant chemotherapy with S‐1 over surgery alone in patients with resected BTCs. The 3‐y OS rate was 77.1% in the S‐1 group and 67.6% in the surgery alone group. In the ITT analysis, the OS of S‐1 was superior to observation, with an HR of 0.694 (95% CI; 0.514–0.935; *P =* 0.008). The 3‐y RFS rate was 62.4% in the S‐1 group and 50.9% in the surgery alone group (HR 0.80, 95% CI; 0.61–1.04, *P =* 0.088). The grade 3 or 4 adverse events associated with the adjuvant S‐1 arm were biliary tract infection (7.2%), diarrhea (2.9%), appetite loss (2.9%), and fatigue (2.9%). The treatment was generally welltolerated. Therefore, adjuvant S1 therapy has become the standard treatment for resected BTCs in Japan and several other Asian countries.

Based on tumor location, a wide variety of invasive surgical resections, including pancreatoduodenectomy or extended hepatectomy, can be applied to BTCs. Care must be taken when administering adjuvant chemotherapy to ensure that the drugs are well‐tolerated and have no adverse effects. In our previous clinical trials, patients with BTCs who underwent major hepatectomy could not tolerate adjuvant GEM chemotherapy compared with those who had other types of surgery, such as pancreatoduodenectomy.^47^, ^48^ In the ASCOT trial, 42 patients underwent major hepatectomy and 165 patients underwent nonmajor hepatectomy in the S‐1 adjuvant chemotherapy group.^49^ The treatment completion proportion was lower in the major hepatectomy group than that in the nonmajor hepatectomy group (59.5% vs 75.8%, *P =* 0.0733), and the median dose intensity was lower in the major hepatectomy group than in the nonmajor hepatectomy group (90% vs 100%, *P =* 0.0358). While surgical procedures are almost identical for other cancers, such as gastric cancer, colorectal cancer, and pancreatic cancer, they are different for BTCs, and therefore BTCs should be managed according to the surgical technique applied.

## 3 ONGOING CLINICAL TRIALS OF ADJUVANT CHEMOTHERAPY

Several ongoing clinical trials have investigated the use of adjuvant chemotherapy for BTCs (Table 2). The ACTICCA‐1 study, a European phase III clinical trial, which aimed to evaluate the efficacy of adjuvant chemotherapy using the GC regimen compared with adjuvant capecitabine is currently ongoing. Other Chinese and Korean phase III trials comparing GEM plus capecitabine with capecitabine alone are also underway. Combination chemotherapy may be a promising standard regimen for adjuvant therapy.

**TABLE 2:** Major ongoing clinical trials of adjuvant and neoadjuvant chemotherapy.

Trial number	Phase	Country or region	Tumor site	Treatment arm	Control arm	Number of patients	Primary endpoint
Adjuvant chemotherapy							
UMIN000036449 (KHBO1901)	II	Japan	ICC, ECC, GBC, AmpC	GC	GS	106	DFS
NCT02170090 (ACTICCA‐1)	III	Europe, Australia	ICC, ECC, GBC	GC	Cap	781	DFS
NCT04401709	III	Korea	ICC, ECC, GBC	GemCap	Cap	490	DFS
NCT03779035	III	China	ICC, ECC, GBC	GemCap	Cap	460	DFS
Neoadjuvant chemotherapy							
NCT01821248 (KHBO1201)	II	Japan	ICC, ECC, GBC, AmpC	GCS		25	Curative resection rate
jRCTs031200388 (JCOG1920)	III	Japan	ICC, ECC, GBC, AmpC	GCS	Upfront surgery	330	OS
NCT03673072 (GAIN)	III	Germany	Incidental GBC, ICC, ECC	GC	Upfront surgery	333	OS

Abbreviations: AmpC, ampulla of Vater carcinoma; Cap, capecitabine; DFS, disease‐free survival; ECC, extrahepatic cholangiocarcinoma; GBC, gallbladder carcinoma; GC, gemcitabine plus cisplatin; GCS, gemcitabine, cisplatin plus S‐1; GemCap, gemcitabine plus capecitabine; GS, gemcitabine plus S‐1; ICC, intrahepatic cholangiocarcinoma; OS, overall survival.

We conducted phase I trials (KHBO1004 and KHBO1202) that reported the feasibility and safety of GC and GS adjuvant chemotherapy in patients with BTCs without major hepatectomy.^50^, ^51^ The recommended GC and GS doses for adjuvant chemotherapy were determined. A randomized phase II clinical trial (KHBO1901) to evaluate the efficacy and safety of adjuvant chemotherapy with GC compared to GS in patients with BTCs without major hepatectomy is currently ongoing.

## 4 NEOADJUVANT CHEMOTHERAPY

Adjuvant chemotherapy has been previously investigated to improve prognosis after surgery. However, some patients cannot complete adjuvant chemotherapy after surgical resection due to postoperative complications or poor tolerance. In the BILCAP study, only 55% of patients completed the eight planned cycles of adjuvant treatment.^45^ In contrast, neoadjuvant chemotherapy offers some advantages, such as (a) downstaging the primary tumors and achieving negative margin; (b) early administration of systemic therapy; (c) high tolerance of systemic therapy; (d) treatment of clinically undetectable microscopic metastatic disease; and (e) avoidance of unnecessary surgery in patients with progressive disease. Neoadjuvant chemotherapy has been considered an effective strategy for improving R0 resection and prognosis in other gastrointestinal cancers, such as pancreatic, esophageal, and gastric cancer.^52^, ^53^, ^54^, ^55^ If the tumors progress, surgical opportunities may be missed. Therefore, the development of neoadjuvant chemotherapy with sufficient tumor control is necessary. To date, no phase III studies have compared neoadjuvant chemotherapy with upfront surgery for potentially resectable BTCs. Recently, the first randomized phase III trial of neoadjuvant chemotherapy with GC vs upfront surgery was reported in Germany.^56^ This study aimed to enroll 333 patients with incidentally detected GBCs after simple cholecystectomy and cholangiocarcinoma. In the neoadjuvant arm, 1000 mg/m^2^ GEM and 25 mg/m^2^ cisplatin were infused on d 1 and 8 and repeated every 3 wk. Treatment with GC was administered for three cycles preoperatively and three cycles postoperatively. In the upfront surgery arm, adjuvant chemotherapy was administered by the investigator. The primary endpoint was OS, and secondary endpoints were QOL, progression‐free survival (PFS), R0 resection rate, toxicity, perioperative morbidity, and mortality.

We conducted a phase III clinical trial (KHBO1401) to evaluate the superiority of GCS over GC in terms of survival in patients with advanced BTC.^18^ GCS demonstrated superior efficacy compared to GC in terms of OS (median OS:13.5 mo with GCS and 12.6 mo with GC; HR 0.79; 90% CI; 0.628–0.996; *P =* 0.046). Objective response rates were 41.5% and 15.0% in the GCS and GC arms, respectively (*P <* 0.001). Therefore, GCS has become the new standard treatment for patients with advanced BTCs. Due to the high response rate, three cases underwent conversion surgery in the GCS arm, while there were no patients who underwent conversion surgery in the GC arm. We also investigated the tumor shrinkage pattern and survival in a subanalysis of the KHBO1401 trial. GCS provided faster and greater tumor shrinkage, with better survival, in comparison with GC. However, 20% of patients showed regrowth after six cycles.^57^ Therefore, GCS in neoadjuvant chemotherapy may achieve a high objective response rate and R0 resection rate in patients with resectable BTCs.

We also conducted a phase II trial (NCT01821248, KHBO1201) to evaluate the efficacy and safety of neoadjuvant chemotherapy using GCS for patients with BTCs and lymph node metastasis using fluorodeoxyglucose positron emission tomography (FDG‐PET). In this study, we employed three cycles of GCS (1000 mg/m^2^ gemcitabine and 25 mg/m^2^ cisplatin on d 1, and oral S‐1 twice a day on d 1–7 every 2 wk) for neoadjuvant chemotherapy in 25 patients. In Japan, a randomized phase III trial (JCOG1920) of neoadjuvant chemotherapy with GCS vs upfront surgery was recently launched. OS was the primary endpoint of this study.

## 5 CONCLUSION

To date, there is no substantial evidence to support the effectiveness of adjuvant chemotherapy for BTCs. However, after the ASCOT trial success, adjuvant chemotherapy with S‐1 has become the current standard therapy. Although the efficacy of neoadjuvant chemotherapy is under investigation, the results of ongoing clinical trials, including adjuvant chemotherapy, are eagerly awaited. Future studies on multimodality therapy are expected to improve the outcomes of patients with BTCs.

## AUTHOR CONTRIBUTIONS

Tatsuya Ioka wrote the initial draft of the article. Yoshitaro Shindo, Makoto Ueno and Hiroaki Nagano contributed to review references and assisted in the presentation of the article. All authors revised and contributed to the interpretation of the findings, and accepted the final article.

## FUNDING INFORMATION

No funding was received for this study.

## CONFLICT OF INTEREST

MU has received research funding from Taiho, Incyte, AstraZeneca, Merck, MSD, Astellas, Eisai, and Ono, and honoraria from Taiho, Yakult, Chugai, Incyte, AstraZeneca, MSD, Eisai, and Ono. Hiroaki Nagano is an editorial member of the Annals of Gastroenterological Surgery. The other authors declare no conflicts of interest.

## ETHICS STATEMENT

Approval of the research protocol: N/A; Informed consent: N/A; Registry and the registration no. of the study/trial: N/A; Animal studies: N/A.
